# Long working hours in the healthcare system of the Belo Horizonte municipality, Brazil: a population-based cross-sectional survey

**DOI:** 10.1186/s12960-017-0203-6

**Published:** 2017-04-21

**Authors:** Juliana M. Andrade, Ada A. Assunção, Mery N. S. Abreu

**Affiliations:** 1 Department of Preventive Medicine, School of Medicine, Federal University of Minas Gerais, Belo Horizonte, Brazil; 2 Department of Applied Nursing, School of Nursing, Federal University of Minas Gerais, Belo Horizonte, Brazil

**Keywords:** Workload, Health personnel, Workplace, Occupational health

## Abstract

**Background:**

Health personnel are key players in developing and improving healthcare systems, caring for individuals and their communities, and helping improve quality of life. However, these professionals are often exposed to long working hours because of the pressing need for their services at potentially any time of day. The long working hours they endure are a major risk factor for both acute and chronic health problems. The present study aimed to analyze occurrences of long working hours and their association with individual characteristics and employment factors among workers in the municipal healthcare system in Belo Horizonte, Brazil.

**Methods:**

In this cross-sectional study, a ramdomly selected proportional sample of 1549 participants was analyzed from among the total of 13 602 workers in the Belo Horizonte municipal healthcare system in 2009. “Long” working hours were defined as >44 h/week. A self-administered questionnaire was used for accumulating data. Associations with outcomes were estimated using logistic regression, in univariate and multivariate models.

**Results:**

The rate of occurrence of long working hours was 31.4% (95% CI 29.1–33.7). Lower educational level (high school, technical, or uncompleted undergraduate [OR 0.60, 95% CI 0.47–0.78 *p <* 0.001], or elementary [OR 0.33, 95% CI 0.19–0.55 *p <* 0.001]) was associated with a lower likelihood of self-reporting long working hours in relation to the group with the highest educational level (completed undergraduate or postgraduate). Male sex (OR 1.62, 95% CI 1.26–2.09 *p <* 0.001), having children (PR 1.54, 95% CI 1.20–1.97 *p =* 0.001), and being in the healthcare provider group (OR 1.82, 95% CI 1.40–2.35 *p <* 0.001) were factors associated with greater likelihood of long working hours.

**Conclusions:**

It was observed that number of long weekly working hours was related to individual characteristics and employment factors.

**Electronic supplementary material:**

The online version of this article (doi:10.1186/s12960-017-0203-6) contains supplementary material, which is available to authorized users.

## Background

Despite the efforts and struggles of labor unions, societal demands, and research that has identified the negative effects of long working hours [1–4] on human health, the worldwide trend is toward increased working hours rather than decreased [5, 6].

In Brazil, there are two million workers in the healthcare system [7]. A 2008 survey of different metropolitan regions found that considerable percentages of health-sector workers had working hours above the legal limit of 44 h/week, especially in the cities of Recife (27.2%), São Paulo (29%), and Salvador (29%). Belo Horizonte, the city where the present study was conducted, recorded a lower 19.2% [8].

For healthcare workers, long working hours have been found to be correlated with health problems such as musculoskeletal disorders [9], psychological disorders [10, 11], daily alcohol consumption [12], being overweight/obese [13], higher chronic fatigue scores and physical discomfort [14], and increased risk of morbidities [2, 15]. Additionally, the longer the working hours, the greater the likelihood that accidents and mistakes will occur within healthcare services [16].

Among healthcare workers in Brazil, it has been observed that excessive work resulting from long working hours leads to dissatisfaction, discouragement, and fatigue [1]. Effort–reward imbalance has also been found associated with the amount of the weekly working hours [17]. Greater length of total working hours (employment hours plus time spent on domestic responsibilities) among female nurses has been found to contribute to harmful behavior with negative health repercussions [18]. Tension, anxiety, insomnia, lack of time for relaxation and leisure activities, and lack of time for taking care of one’s home and children have also been identified among hospital-sector nurses [19].

In short, long working hours have been found correlated with morbidity among healthcare workers. However, knowledge gaps still exist and study results are not always aligned with each other [20, 21]. Thus, identifying the individual characteristics and employment factors of healthcare workers according to their exposure to long working hours is important, because this information is useful for drawing up human resources policies within the healthcare system.

The aim of this study was to analyze occurrences of long weekly working hours and their association with individual characteristics and employment factors among workers in the Belo Horizonte municipal healthcare system.

## Methods

### Data and study population

A cross-sectional study was conducted among workers in the Belo Horizonte municipal healthcare system in 2009. All workers in this sector, regardless of employment status (permanent, temporary, or intern), who were working at the randomly selected facilities were eligible. This particular healthcare system encompasses different occupations and functions that can be classified, according to World Health Organization criteria [22] as healthcare providers and management and support. Healthcare providers are workers whose objective is to promote or restore health (e.g., doctors, nurses, nursing assistants, nutritionists, psychologists, physiotherapists, and speech therapists). Management and support are workers within administration, information, cleaning, and other activities that facilitate good functioning of the healthcare units. The total number of contracted hours comprising working hours varied among the above occupations. For physicians, social workers, and physiotherapists/occupational therapists, contracted working hours were 24, 30, and 36 h/week, respectively. For other occupations, for example nurses, regulations in force for general workers apply, i.e., 44 h/week. The sample size was estimated at 2205 workers, taking into consideration the following parameters: total number of workers in the sector (13 602), 50% rate of occurrence of long working hours, 5% significance level, 2.5% precision, and a 50% increase in the sample size to offset errors in filling out questionnaires, as well as refusals and losses [23]. A total of 2205 workers were invited to participate in the study, of which 1808 complied. Among these, 259 did not satisfy the study protocol for the variable of interest “long working hours”. Thus, there was a total loss of 656 participants. The final sample of this study comprised 1549 participants (response rate 70.2%). Participants were selected based on stratified proportional random sampling according to geographical area, level of complexity of care provided (healthcare centers, specialist outpatient clinics, emergency units, or district management), and occupation. Proportional distribution of workers per stratum was based on the list of employees supplied by the respective human resources department. The sample included both part-time and full-time workers. Participants were selected via random numbers generated through Epi-Info 3.5.3 software tools, (Atlanta, GA, USA). Eight pilot studies were performed at several health facilities to evaluate and adjust the instrument. Each pilot study involved about 20 respondents who were, therefore, excluded from the randomization. Information was obtained using a paper self-administered questionnaire [24], accompanied by one of nine trained interviewers who could answer any queries. Up to three attempts were initially made to locate the randomly selected worker before he or she was considered lost from the study. Additional draws were conducted, respecting the proposed stratification criteria, to replace any workers who were selected but were absent because of vacation, transfer, retirement, or death. The study was approved by the ethics committees of the Federal University of Minas Gerais (542/07) and the city authorities of Belo Horizonte (054/06), and observed the ethical principles of the Declaration of Helsinki [25]. Written informed consent was obtained.

### Variables

In the present study, the dependent variable, long working hours, was defined as >44 h/week, which is the limit set forth in the Brazilian constitution [26].

This variable was assessed based on the question, “How many income-earning hours do you work during 1 week? ___ hours/week.” This question encompassed both the main job and any supplemental employment. For analysis purposes, the categories of ≤44 and >44 h were established.

The covariates considered relation either to individual characteristics (sex, age, marital status, children, and educational level) or to employment characteristics (occupational group and income).

“Educational level” was classified into three categories: completed undergraduate or graduate level; high school, technical, or uncompleted undergraduate levels; and elementary education level. The workers were distributed into two occupational groups: healthcare providers, and management and support [22]. Income and age were evaluated in two categories defined by means of the median cutoff point: >US$ 432.90 equivalent and ≤US$ 432.90 per month; ≤42 and ≥43 years old, respectively. The variables income and age were categorized by the median to obtain homogenous groups for analyses.

### Statistical analyses

STATA 12.0 (StataCorp LP, College Station, Texas, USA) was used for statistical analyses, while univariate and multivariate analyses were conducted. The magnitude of the association between exposure and outcome was estimated via odds ratio (OR), using a binary logistic model. Multivariate analysis was conducted in a single block that took individual and employment characteristics into consideration. The backward elimination modeling method was used. Significance level was taken to be 5% and the confidence interval was 95%. Possible interactions between the variables in the final model were tested, and the goodness of fit was tested using the Hosmer–Lemeshow test [27, 28].

## Results

Table 1 shows the distribution of the sample of municipal healthcare workers in accordance with their self-reported working hours, and Table 2 shows the individual and employment characteristics. It was found that 31.4% (95% CI 29.1–33.7) of the respondents self-reported long working hours. Women comprised 69.5% of the population studied. The population studied was predominantly composed by female (69.5%), by participants married (53.8%), with having children (63.1%), and with high school, technical, or uncompleted undergraduate educational level (49.9%). The participants’ ages ranged 16–73 (median 42) years old. In relation to employment characteristics, approximately 54% were in the healthcare provider group and 54.3% had monthly income >US$ 432.90 equivalent (Tables 1 and 2).

**Table 1:** Distribution of the sample of municipal healthcare workers according to working hours (sample considered = 1549) and individual characteristics

			Long working hours		
Variable	Total *N*	Prevalence *N* (%)	OR	95% CI	*p* value
Sex					
Females	1070 (69.5)	305 (28.5)	1.0	–	
Males	469 (30.5)	179 (38.2)	1.55	1.23–1.95	<0.001
Total	1539	484			
Age group (years)					
≤42	774 (50.5)	254 (32.8)	1.0	–	
≥43	760 (49.5)	229 (30.1)	0.88	0.71–1.09	0.258
Total	1534	483			
Marital status					
Single	713 (46.2)	211 (29.6)	1.0	–	
Married	832 (53.8)	274 (32.9)	1.17	0.94–1.45	0.159
Total	1545	485			
Children					
No	569 (36.9)	160 (28.1)	1.0	–	
Yes	970 (63)	323 (33.3)	1.28	1.02–1.60	0.035
Total	1539	483			
Educational level					
Completed undergraduate or graduate level	639 (41.6)	262 (41)	1.0	–	
High school, technical, or uncompleted undergraduate levels	766 (49.9)	195 (25.5)	0.49	0.39–0.62	<0.001
Elementary education level	131 (8.5)	24 (18.3)	0.32	0.20–0.52	<0.001
Total	1536	481			

*OR* odds ratio, *CI* confidence interval

**Table 2:** Distribution of the sample of municipal healthcare workers according to working hours (sample considered = 1549) and employment characteristics

			Long working hours		
Variable	Total *N*	Prevalence *N* (%)	OR	95% CI	*p* value
Occupational group					
Management and support	652 (46.4)	148 (22.7)	1.0	–	
Providers	753 (53.6)	298 (39.6)	2.23	1.76–2.82	<0.001
Total	1405	446			
Income^a^					
>US$ 432.9	777 (54.3)	280 (36)	1.0	–	
≤US$ 432.9	653 (45.7)	161 (24.7)	0.58	0.46–0.73	<0.001
Total	1430	441			

*OR* odds ratio, *CI* confidence interval

^a^Minimum salary at the time US$ 201.29

Long working hours were more common among males (38.2%) than females (28.5%); among those with children (33.3%) than those without (28.1%); among those who completed undergraduate or graduate education (41%) than those with high school, technical, or uncompleted undergraduate education (25.5%) or those only with elementary education (18.3%); among healthcare providers (39.6%) than management and support workers (22.7%); and among workers with monthly income >US$ 432.90 (36%) than those with ≤US$ 432.90 (24.7%) (Tables 1 and 2). The variables selected for multivariate analysis (*p <* 0.20) were sex, marital status, children, education, healthcare providers, and income.

Table 3 presents the factors that, according to the final model, remained associated with working hours with *p <* 0.05: sex, having children, education, and healthcare workers. The model presented a good fit, according to Hosmer–Lemeshow test (*p =* 0.352).

**Table 3:** Final adjusted model for factors associated with long working hours in the municipal healthcare workers (sample considered = 1380)

Variable	Long working hours		
	OR	95% CI	*p* value
Sex			
Females	1.0	–	
Males	1.62	1.26–2.09	<0.001
Children			
No	1.0	–	
Yes	1.54	1.20–1.97	0.001
Educational level			
Completed undergraduate or graduate level	1.0	–	
High school, technical, or uncompleted undergraduate levels	0.60	0.47–0.78	<0.001
Elementary education level	0.33	0.19–0.55	<0.001
Occupational group			
Management and support	1.0	–	
Providers	1.82	1.40–2.35	<0.001

Hosmer–Lemeshow test *p =* 0.352

*OR* odds ratio, *CI* confidence interval

Lower educational level (high school, technical, or uncompleted undergraduate [OR 0.60, 95% CI 0.47–0.78 *p <* 0.001], or elementary [OR 0.33, 95% CI 0.19–0.55 *p <* 0.001]) was associated with a lower likelihood of self-reporting long working hours in relation to the group with the highest educational level (completed undergraduate or postgraduate). Male sex (OR 1.62, 95% CI 1.26–2.09 *p <* 0.001), having children (PR 1.54, 95% CI 1.20–1.97 *p =* 0.001) and being in the healthcare provider group (OR 1.82, 95% CI 1.40–2.35 *p <* 0.001) were factors associated with greater likelihood of long working hours.

## Discussion

This study aimed to identify occurrences of long working hours among a sample of municipal workers in Belo Horizonte. Long working hours were predominantly observed among the male workers, individuals with higher educational levels, and those who had children, and in the group with occupations that provided healthcare services, i.e., among workers who interacted directly with healthcare service users. Over 30% of the respondents stated they worked >44 h/week, and this was greater than for the general proportion (19.2%) found in Belo Horizonte in the same year [8]. This reality within the healthcare system is not surprising [3] and is probably related with the nature of the tasks involved, which require continual attention, and also with multiple employment strategies, as analyzed below.

Greater occurrence of long working hours was observed among males than females, and this result aligns with national statistics from the Netherlands [29], Norway [30], and the United Kingdom [31]. In Australia, the mean number of weekly working hours among male doctors (48) was found to exceed that of female doctors (37.1) [2]. In Brazil, studies conducted among nurses [17, 18] also showed longer working hours among men than women.

Men and women participate in the labor market in different ways. Economic participation indicators for Brazil reveal that, over the period of 1993–2005, the female economically active population increased from 28 million to 41.7 million, activity rate increased from 47 to 53%, and percentage of women within the overall workforce increased from 39.6 to 43.5% [32]. Despite these considerable increases, the activity rates for women continue to be notably lower than those for men. Number of working hours is another variable that expresses differences when the profile of the Brazilian workforce is analyzed. Despite advances in professions and roles, traditional family models are continuing, which may explain the sex-related differences in the number of the working hours within employment [33]. In other words, these working hours are longer for men, while their domestic working hours are shorter. For women, working hours within employment are shorter, but domestic working hours are longer [17].

The results from the present study showed an association between long working hours and having children. This result is also not surprising because the presence of children gives rise to financial and family responsibilities [34]. However, caution is needed in evaluating life situations involving children, because domestic demands may reduce available time for sleep and self-care, especially for women [18].

Shorter working hours predominated in the group of individuals with lower educational levels, and this result was statistically significant (*p <* 0.05). In the Netherlands [29] and the United Kingdom [35], the groups with the highest educational level accounted for the greatest proportion of long working hours. In Korea, the result was different: among workers who reported excessively long working hours (≥60 h/week), professionals with lower educational levels predominated [36]. Among women in Norway, no association between long working hours and educational level was observed. Among men, the group that stated that their working week was 41–48 h included more with higher educational levels than did the reference group (35–40 h/week). However, in the group that declared an even longer working week, there was a higher proportion of workers with lower educational levels [30], similar to Korea [36].

In the Brazilian population overall, individuals with higher educational levels were found to have shorter weekly working hours (31–40) than those with lower educational levels (41–44) [37]. This same tendency was seen in Korea [36] and Norway [30], although the data indicating a relationship between lower educational level and long working hours in those two countries referred to ≥60 working hours/week.

In Brazil, formal employment contracts and higher educational levels are found to be directly related to shorter working hours. In general, longer weekly working hours (41–44) are performed by individuals with lower educational levels, but the results from the present study diverge from this pattern. Despite the difficulties in comparing Brazilian data and data from other countries (the reference category in the present study was ≥44 h/week), it is likely that the nature of the tasks involved in the sample analyzed herein (which require a higher education level) provides an explanation why this group works more hours per week than the group with lower educational levels. This higher educational level group included most of the workers (healthcare providers) who cared directly for the users. Care activities are generally organized based on duty rosters that exceed the traditional 8 working hours/day, and this is more common than in the management and support workers group. For this reason, the association between the healthcare providers group and long working hours could be expected. Moreover, it is known that multiple employment are an extension mode of working time that has become common for this group [3]. It is worth emphasizing that the effects of the number of working hours may become intensified, depending on the characteristics of the time organization of the work [17].

In Belo Horizonte, the number of individuals working in healthcare services who worked more than one job increased by 54.5% between 1998 and 2008 [8]. In Rio de Janeiro, 41.2% of hospital nurses stated that they had more than one employment contract [19]. Additionally, >50% of the healthcare workers in hospital wards in Rio Grande do Norte [1] and São Paulo [17] undertook multiple employment to supplement their family income.

Paradoxically, in the present study, “income” did not remain associated with long working hours. This discrepancy can be explained by the fact that the variable income is correlated with the variable education. To clarify this result, a model without the variable education was tested. By using this strategy, the variable income remained associated with long working hours (result not presented). Higher income was likely possible because of workers holding multiple jobs, but it was not possible to move further with these hypotheses in this setting because of the limits of the study design.

Cross-sectional studies introduce bias (especially with regard to selection) and problems with regard to testing causal hypotheses, and these factors need to be taken into consideration when interpreting the results. Regarding limits related to data-gathering strategies, it should be kept in mind that information obtained from questionnaires comprises indirect measurements, given that it is based on self-reporting. It thus depends on memory and on the clarity of the questions, and may be influenced by particular interests of the interviewees [38]. The lack of consensus in the literature regarding the definition of long working hours also limits the strength of comparisons [39]. These comparisons are made difficult because the ranges for numbers of working hours are not always the same. Furthermore, studies have focused on other sectors or else investigated samples of doctors and nurses, rather than the sector in an overall manner. One further limitation was the loss of information relating to each variable analyzed.

Nonetheless, all losses were <10%. The advantage of the approach used here, with a considerably large sample of healthcare workers that included different professional categories in an array of geographical areas and with all types of services provided by the municipal sector, needs to be emphasized. The advantage of the random nature of the sample is also a strength, along with the replacement of the selected individuals who were not found at their workplace after three attempts, which was done while still respecting the geographical area, level of complexity of the healthcare service provided, and occupational group. In other words, measures to minimize possible sources of selection bias (such as loss of workers from a specific group) were adopted. Moreover, the description of occurrences of long working hours and their distribution within the sample identified group characteristics according to the highest and lowest occurrences of the event of interest. This study thus provides important clarification regarding the sociodemographic and occupational profiles of healthcare workers in accordance with their exposure to long working hours. This information is useful for drawing up human resources policies within the healthcare system. The study report adhered to the Strengthening the Reporting of Cross-sectional Studies in Epidemiology (STROBE) guidelines (Additional file 1).

## Conclusions

It was observed that the number of long weekly working hours was related to individual characteristics (male, highest educational level, having children) and to the employment factors (healthcare providers). These results, though they are exploratory in nature, offer a useful perspective for work administration that serves to strengthen human resources within the healthcare provision industry.

## Additional files

Additional file 1: STROBE Statement—Checklist of items that should be included in reports of cross-sectional studies. (PDF 83.5 kb)

Additional file 2: Free and informed consent statement. (PDF 383 kb)

Additional file 3: Ethics committees of the Federal University of Minas Gerais (542/07). (PDF 273 kb)

Additional file 4: City authorities of Belo Horizonte (054/06). (PDF 278 kb)

## Abbreviation

SUS: Sistema Único de Saúde—Brazilian National Healthcare System
