# Supplementary material for: Long working hours in the healthcare system of the Belo Horizonte municipality, Brazil: a population-based cross-sectional survey
Source: Hum Resour Health. 2017 Apr 21;15:30. doi: 10.1186/s12960-017-0203-6 (PMC5399831; doi:10.1186/s12960-017-0203-6)
Supplement: Supplementary file 2 — Free and informed consent statement. (PDF 383 kb) [file 12960_2017_203_MOESM2_ESM.pdf]

Emprego, trabalho e saúde no setor saúde

TERMO DE CONSENTIMENTO

Estudo quantitativo

Estamos convidando você a participar de uma pesquisa que será realizada na Faculdade de Medicina da UFMG. A pesquisa **emprego, trabalho e saúde no setor saúde** pretende descrever as características ocupacionais e sócio-demográficas dos profissionais da Secretaria Municipal de Saúde de Belo Horizonte, sorteados aleatoriamente. Caso concorde em participar desta pesquisa, você deverá responder ao questionário de caráter confidencial e individual. Sua participação neste estudo é voluntária e você poderá não concordar em fazer parte da nossa amostra sem que isto traga qualquer prejuízos no seu trabalho.

Todas as informações obtidas serão guardadas em segurança pelo pesquisador, sendo tais informações sigilosas. Você será identificado por um número e seu nome não será divulgado. Todos os dados obtidos serão utilizados exclusivamente para esta pesquisa cientifica e somente terão acesso a eles os pesquisadores envolvidos no projeto. Seu nome não será identificado em nenhuma publicação que resultar deste estudo

Caso tenha dúvidas ou necessite obter outras informações, favor entrar em contato com Maria Núbia Alves Cruz (3409-9711) ou Ada Ávila Assunção (3409-9815). Você também poderá entrar em contato com o Comitê de Ética e Pesquisa da Prefeitura Municipal de Belo Horizonte no endereço Avenida Afonso Pena, 2336 - 9º andar, Bairro Funcionários, ou pelo telefone 3277-5309; ou com o Comitê de Ética e Pesquisa da Universidade Federal de Minas Gerais no endereço Av. Antônio Carlos, 6627, Unidade Administrativa II, 2 ° andar, Campus Pampulha, ou pelo telefone (031) 3499-4592.

Consentimento:

Declaro que li e entendi todas as informações contidas neste Termo de Consentimento, que concordo com as propostas aqui descritas e que recebi uma cópia do mesmo com a minha assinatura. Decido participar da pesquisa **emprego, trabalho e saúde no setor saúde** respondendo ao questionário, ciente de que posso negar a minha participação no momento que desejar.

Local e data:

Nome:

Assinatura:

Assinatura do responsável pela pesquisa:
