# Supplementary material for: Long working hours in the healthcare system of the Belo Horizonte municipality, Brazil: a population-based cross-sectional survey
Source: Hum Resour Health. 2017 Apr 21;15:30. doi: 10.1186/s12960-017-0203-6 (PMC5399831; doi:10.1186/s12960-017-0203-6)
Supplement: Supplementary file 3 — Ethics committees of the Federal University of Minas Gerais (542/07). (PDF 273 kb) [file 12960_2017_203_MOESM3_ESM.pdf]

	Universidade Federal de Minas Gerais<br>*Comitê de Ética em Pesquisa da UFMG - COEP*

Parecer nº. ETIC 542/07

Interessado(a): Profa. Ada Ávila Assunção
Departamento de Medicina Preventiva e Social
Faculdade de Medicina-UFMG

DECISÃO

O Comitê de Ética em Pesquisa da UFMG – COEP aprovou, no dia 28 de novembro de 2007, o projeto de pesquisa intitulado **"Condições de emprego, condições de trabalho e saúde dos trabalhadores da saúde"** bem como o Termo de Consentimento Livre e Esclarecido.

O relatório final ou parcial deverá ser encaminhado ao COEP um ano após o início do projeto.

Profa. Maria Teresa Marques Amaral
Coordenadora do COEP-UFMG

Av. Pres. Antonio Carlos, 6627 – Unidade Administrativa II - 2º andar – Sala 2005 – Cep31270-901 – BH-MG
Telefone: (031) 3499-4592- FAX: (031)3499-4516 - e-mail: coep@prpq.ufmg.br
