# Supplementary material for: Long working hours in the healthcare system of the Belo Horizonte municipality, Brazil: a population-based cross-sectional survey
Source: Hum Resour Health. 2017 Apr 21;15:30. doi: 10.1186/s12960-017-0203-6 (PMC5399831; doi:10.1186/s12960-017-0203-6)
Supplement: Supplementary file 4 — City authorities of Belo Horizonte (054/06). (PDF 278 kb) [file 12960_2017_203_MOESM4_ESM.pdf]

PREFEITURA BH

COMITÊ DE ÉTICA EM PESQUISA-SECRETARIA MUNICIPAL DE SAÚDE DE BELO HORIZONTE (CEP-SMSA/PBH)

Avaliação de projeto de pesquisa – **Protocolo 054/2006**

Projeto: Condições de Emprego. Condições de Trabalho e Saude dos Trabalhadores de Saude"

Pesquisadora: Maria Cristina Ramos de Vasconcellos

Pesquisador Responsável

1) Ada Avila Assunção (coordenação)
2) Soraya Almeida Belisário (coordenação)
3) Luciana Souza d'Avila (*Bolsista do Projeto*)
4) Maria Cristina Ramos de Vasconcellos Coelho (Mestranda / Programa de Pos-graduação em Saude Publica/UFMG)
5) Vivian Patricia Andrade de Souza (*Bolsista do Projeto*)
6) Juliana da Costa Martins

O projeto acima referido cumpriu os requisitos da resolução 196/96 da CONEP, tendo sido aprovado na reunião do Comitê de Ética em Pesquisa da Secretaria Municipal de Saude de Belo Horizonte.

O relatorio final ou parcial devera ser encaminhado ao CEP um ano apos inicio do projeto ou ao final desde, se em prazo inferior a um ano

Celeste de Souza Rodrigues
Coordenadora do CEP-SMSA/PBH

Belo Horizonte, 01 de dezembro de 2006
